# Identification of Susceptibility Modules and Genes for Cardiovascular Disease in Diabetic Patients Using WGCNA Analysis

**DOI:** 10.1155/2020/4178639

**Published:** 2020-05-10

**Authors:** Weiwei Liang, Fangfang Sun, Yiming Zhao, Lizhen Shan, Hanyu Lou

**Affiliations:** ^1^Department of Endocrinology, The Second Affiliated Hospital, Zhejiang University School of Medicine, Hangzhou, China; ^2^Department of Colorectal Surgery, The Second Affiliated Hospital of Zhejiang University School of Medicine, China; ^3^Cancer Institute (Key Laboratory of Cancer Prevention and Intervention, China National Ministry of Education, Key Laboratory of Molecular Biology in Medical Sciences), The Second Affiliated Hospital, Zhejiang University School of Medicine, China

## Abstract

**Objective:**

To identify susceptibility modules and genes for cardiovascular disease in diabetic patients using weighted gene coexpression network analysis (WGCNA).

**Methods:**

The raw data of GSE13760 were downloaded from the Gene Expression Omnibus (GEO) website. Genes with a false discovery rate < 0.05 and a log2 fold change ≥ 0.5 were included in the analysis. WGCNA was used to build a gene coexpression network, screen important modules, and filter the hub genes. Gene Ontology (GO) and Kyoto Encyclopedia of Genes and Genomes (KEGG) pathway enrichment analyses were performed for the genes in modules with clinical interest. Genes with a significance over 0.2 and a module membership over 0.8 were used as hub genes. Subsequently, we screened these hub genes in the published genome-wide SNP data of cardiovascular disease. The overlapped genes were defined as key genes.

**Results:**

Fourteen gene coexpression modules were constructed via WGCNA analysis. Module greenyellow was mostly significantly correlated with diabetes. The GO analysis showed that genes in the module greenyellow were mainly enriched in extracellular matrix organization, extracellular exosome, and calcium ion binding. The KEGG analysis showed that the genes in the module greenyellow were mainly enriched in antigen processing and presentation, phagosome. Fifteen genes were identified as hub genes. Finally, *HLA-DRB1*, *LRP1*, and *MMP2* were identified as key genes.

**Conclusion:**

This was the first study that used the WGCNA method to construct a coexpression network to explore diabetes-associated susceptibility modules and genes for cardiovascular disease. Our study identified a module and several key genes that acted as essential components in the etiology of diabetes-associated cardiovascular disease, which may enhance our fundamental knowledge of the molecular mechanisms underlying this disease.

## 1. Introduction

Cardiovascular disease is a common public health problem that occurs in individuals over 45 years of age. Diabetes mellitus (DM) is an independent risk factor for cardiovascular disease [1]. The Framingham study showed that patients with diabetes have a twofold to fourfold higher risk of developing cardiovascular disease than those without diabetes [2]. Clinically, patients with cardiovascular disease and diabetes mellitus generally have a poorer prognosis compared with the patients without diabetes mellitus [3]. Larger necrotic cores of plaques and a higher incidence of healed plaque ruptures within the coronary arteries were observed in patients with DM compared with those without DM [4]. Cardiovascular disease is a major cause of death in patients with diabetes.

The mechanisms underlying the DM-associated progression of cardiovascular disease are not fully understood. There are several potential mechanisms through which diabetes causes the acceleration of atherosclerosis [5]. Clinically, patients with diabetes may also have hypertension, abnormalities of lipid metabolism, and insulin resistance; all of which are linked to an increased cardiovascular risk [6]. At the systemic level, hyperglycemia, oxidative stress, and inflammation may promote cardiovascular disease in patients with diabetes [7]. Conversely, at the cellular or molecular levels, little is known about this phenomenon. Therefore, we aimed to detect the genetic basis of cardiovascular disease between patients with and without diabetes.

Weighted gene coexpression network analysis (WGCNA) is a bioinformatics analytical method that is used frequently to explore effectively the relationships between genes and phenotypes [8]. The distinct advantage of WGCNA is that it can cluster genes into coexpression modules and build a bridge between sample characteristics and changes in gene expression. WGCNA analyzes thousands of genes, identifies gene modules that are relevant to clinically characters, and, finally, identifies key genes in the disease pathways, for further validation. WGCNA provides a systems-level insight into the signaling networks that may be associated with a phenotype of interest.

In this study, we aimed to identify diabetes-associated susceptibility modules and genes for cardiovascular disease. We used the rich data from the GEO database. WGCNA was used to build a gene co-expression network, to screen important modules, and to filter the key genes. This paper provides novel insights that will help understand the molecular mechanism of cardiovascular disease in patients with diabetes.

## 2. Methods

### 2.1. Data Sources and Searches

We took the full use of the Gene Expression Omnibus (GEO) database, which represents the largest resource of public microarray data. We searched the GEO for high-throughput functional genomics experiments of type 2 diabetes. This study included several selection criteria for data selection, as follows: (1) the samples included blood vessel samples in diabetic and nondiabetic patients, (2) the study type was narrowed down to expression profiling by array, (3) the organism was restricted to Homo sapiens, (4) the raw data or processed data were public and accessible, and (5) the total sample size was larger than 15. The gene expression profiles of GSE13760 were selected because it exhibited the best quality and the most appropriate sample size for performing WGCNA analysis.

### 2.2. Data Download and Statistical Data Analysis

The raw data of GSE13760 were downloaded from the GEO website. The limma package was used to perform quality control, preprocessing, and statistical data analysis. The robust multi-array average (RMA) method was used to normalize data. A limma analysis was used to identify genes with a false discovery rate < 0.05 and a log2 fold change ≥ 0.5 for WGCNA analysis.

### 2.3. WGCNA Network Construction and Module Identification

We used the WGCNA R package to construct the coexpression network [8]. First, samples were clustered to assess the presence of any obvious outliers. Second, the automatic network construction function was used to construct the coexpression network. The R function pickSoftThreshold was used to calculate the soft thresholding power *β*, to which coexpression similarity is raised to calculate adjacency. Third, hierarchical clustering and the dynamic tree cut function were used to detect modules. Fourth, gene significance (GS) and module membership (MM) were calculated to relate modules to clinical traits. The corresponding module gene information was extracted for further analysis. Finally, we visualized the network of eigengenes.

### 2.4. Functional Enrichment Analysis

Genes in modules of interest were extracted for further functional enrichment analysis. A Gene Ontology analysis (GO) was used to identify characteristic biological attributes [9]. A Kyoto Encyclopedia of Genes and Genomes (KEGG) pathway enrichment analysis was performed to identify functional attributes [10]. Significance was set at *P* < 0.05.

### 2.5. Identification of Key Genes

We defined the genes with a GS over 0.2 and an MM over 0.8 in the clinically relevant gene module networks as hub genes. Subsequently, we screened these hub genes in the published genome-wide SNP data of cardiovascular disease. The overlapping genes were defined as key genes. A classical *t*-test was performed to compare the differences in the expression of key genes between the two groups using a *P* value < 0.05 to indicate statistical significance. The ggplot2 R package was used to draw violin plots of the expression of key genes.

## 3. Results

### 3.1. Data Collection

We downloaded the gene chip GSE13760 of arterial tissues together with its clinical manifestation data from the GEO database. The dataset was platform was GPL571 (Affymetrix Human Genome U133A 2.0 Array). There were 10 type 2 diabetic blood vessel samples and 11 control blood vessel samples. The raw data had been processed, and the gene expression matrix provided by the website was directly used in the analysis.

**Figure 1:**
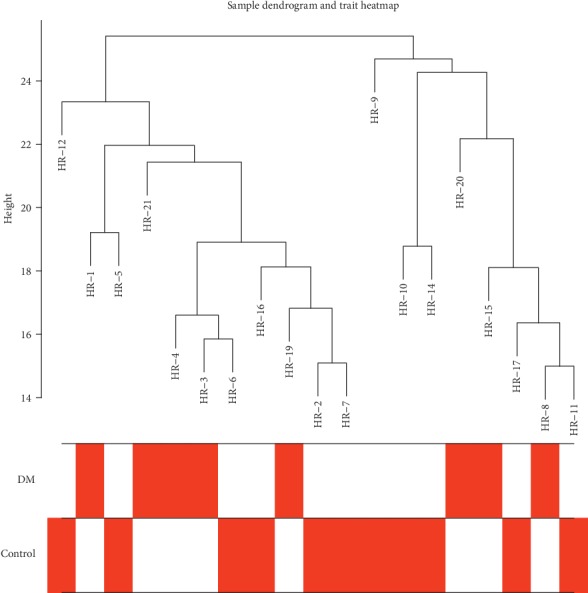
Clustering dendrogram of samples based on their Euclidean distance.

The raw data were normalized using the RMA method in the limma package. Genes with a false discovery rate < 0.05 and a log2 fold change ≥ 0.5 were included in the WGCNA analysis. First, we checked for genes and samples with too many missing values, and all genes passed the cut-off values. Next, we clustered the samples, to identify if there are any obvious outliers. The height cut-off value was set at 30, and all samples were included in our analysis (Figure 1).

### 3.2. Construction of Gene Coexpression Modules

**Figure 2:**
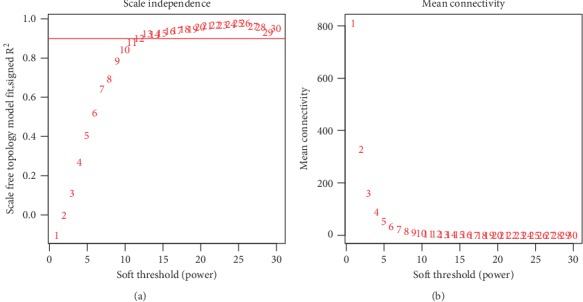
Analysis of network topology for various soft-thresholding powers. (a) The *x*-axis reflects the soft-thresholding power. The *y*-axis reflects the scale-free topology model fit index. (b) The *x*-axis reflects the soft-thresholding power. The *y*-axis reflects the mean connectivity (degree).

To construct a WGCNA network, we first calculated the soft thresholding power *β*, to which the coexpression similarity is raised to calculate adjacency. We used of the function pickSoftThreshold function in WGCNA, which performs the analysis of network topology analysis. The soft thresholding power *β* was set at 11 in the subsequent analysis, because the scale independence reached 0.9 (Figure 2(a)) and had a relatively high-average connectivity (Figure 2(b)).

**Figure 3:**
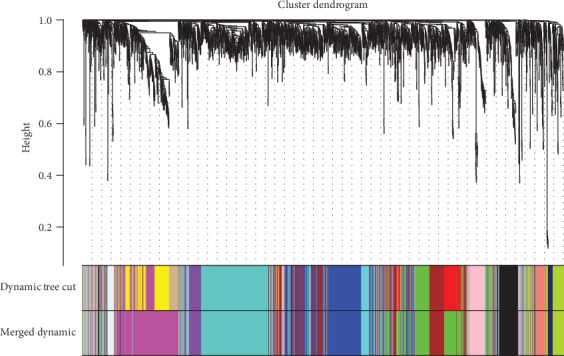
Clustering dendrogram of genes, with dissimilarity based on topological overlap, together with assigned module colors.

We constructed the gene network and identified modules using the one-step network construction function of the WGCNA R package. To cluster splitting, the soft thresholding power was set at 11, the minimum module size was set at 30, and the deepSplit was set at 2 (which implies a medium sensitivity). Finally, 14 gene coexpression modules were finally constructed (Figure 3).

### 3.3. Analysis of the Relationship between Pairwise Gene Coexpression Modules and Eigengenes

**Figure 4:**
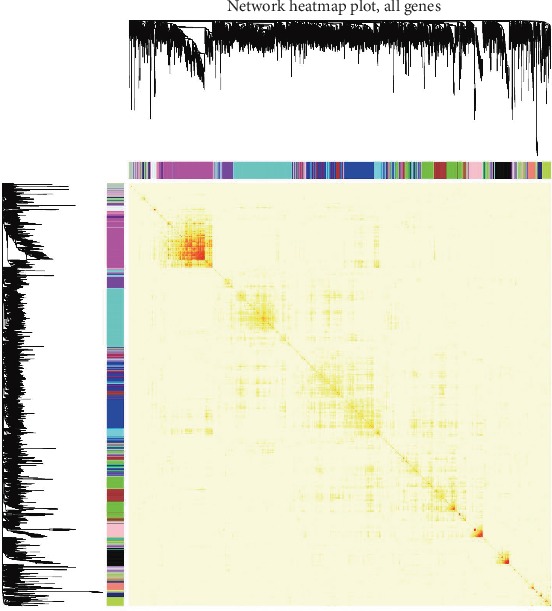
Visualization of the WGCNA network using a heatmap plot. The heatmap depicts the topological overlap matrix (TOM) among all modules included in the analysis. The light color represents a low overlap, and the progressively darker red color represents an increasing overlap.

We mapped the relationships between the identified modules (Figure 4). The heatmap depicts the topological overlap matrix (TOM) among all genes included in the analysis. The light color represents a low overlap, and the progressively darker red color represents an increasing overlap. The results of this analysis indicated that the gene expression was relatively independent between modules.

**Figure 5:**
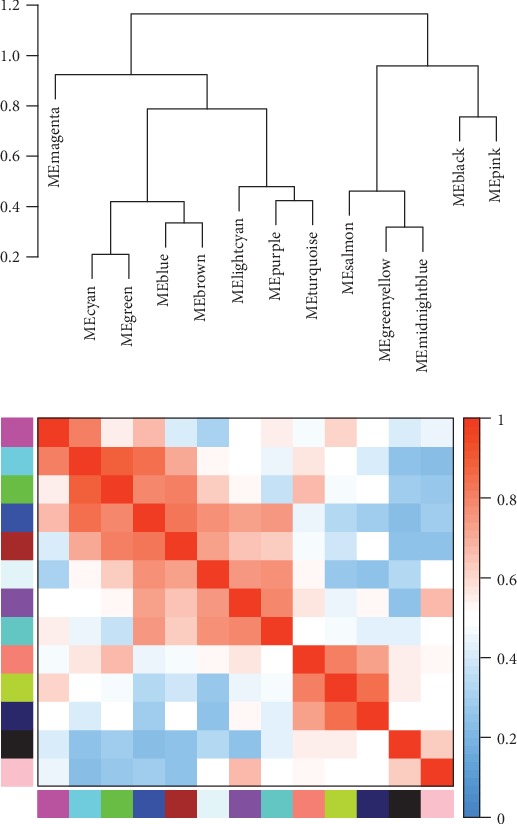
Eigengene dendrogram and eigengene adjacency plot.

We analyzed the connectivity of eigengenes. Eigengenes can provide information about the relationship between pairwise the gene coexpression modules. We clustered the eigengenes. The results showed that 14 modules could be clustered into two clusters (Figure 5), and four combinations (modules cyan and green, modules blue and brown, modules purple and turquoise, and modules greenyellow and midnightbule) had a high degree of interaction connectivity.

### 3.4. Identification of Key Modules

**Figure 6:**
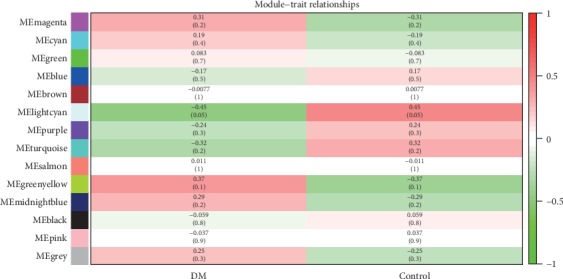
Module–trait associations. Each row corresponds to a module, and each column corresponds to a trait. Each cell contains the corresponding correlation and *P* value. The table is color-coded by correlation according to the color legend.

We correlated modules with clinical characteristics and searched for the most significant associations. The results of this analysis showed that module greenyellow was mostly significantly correlated with diabetes (Figure 6).

### 3.5. Functional Analysis of the Key Module

**Figure 7:**
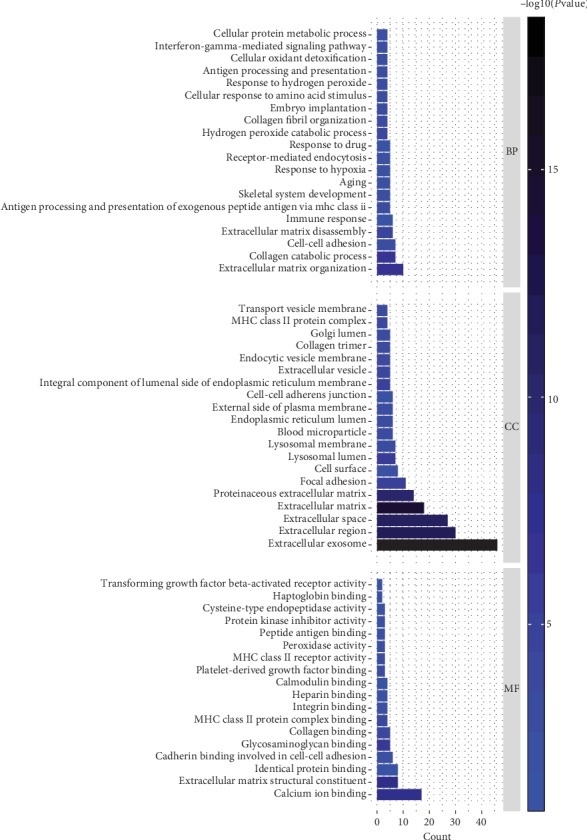
Gene Ontology analysis of the genes involved in the greenyellow module regarding biological process, cellular component, and molecular function.

**Figure 8:**
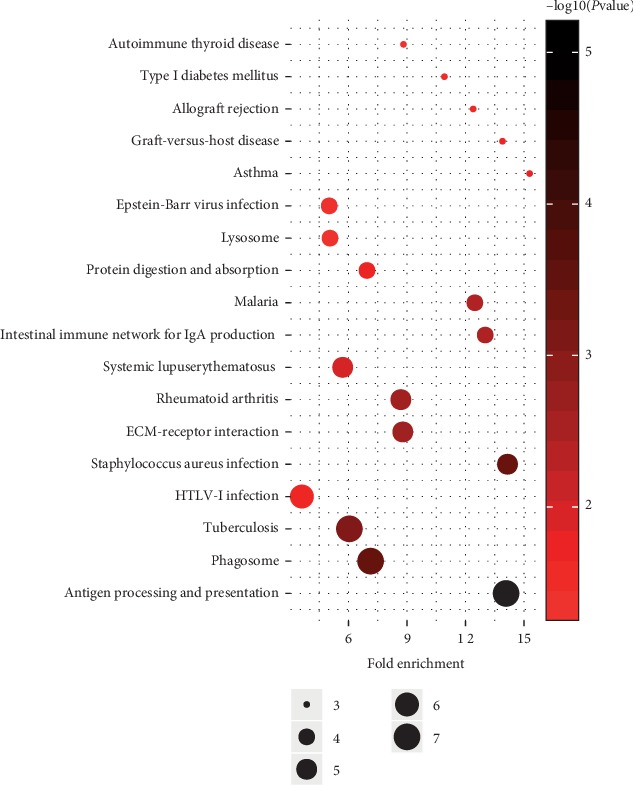
KEGG analysis of genes involved in lightyellow module. The node size reflects the gene count, and the node color reflects the *P* value [−log10 (*P* value)].

We conducted a GO analysis and KEGG analysis of genes in the module greenyellow (Figures 7 and 8). The results of these analyses showed that, regarding the biological process, the genes were mainly enriched in extracellular matrix organization. As for the cellular component, the genes were mainly enriched in extracellular exosome. Finally, regarding molecular function, the genes were mainly enriched in calcium ion binding.

We then performed a functional analysis (KEGG analysis) of the genes in the greenyellow module and identified the module-regulated pathway. The result of this analysis showed that the greenyellow module-regulated pathways included antigen processing and presentation, phagosome.

### 3.6. Identification of Key Genes

**Figure 9:**
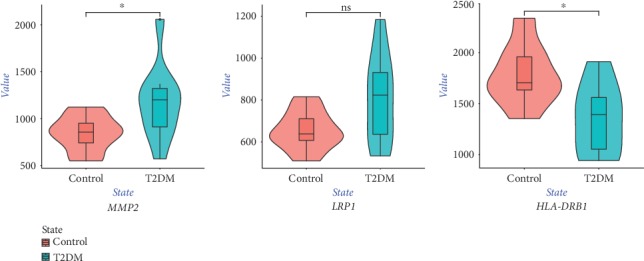
Violin plot of the expression level of key genes. The red violin reflects the control group, and the green violin reflects the T2DM group. ^∗^*P* < 0.05.

The greenyellow module contained 78 genes. Using a GS over 0.2 and an MM over 0.8 as cut-off criteria, 15 genes were identified as hub genes (Table 1). Subsequently, we screened these hub genes in the published cardiovascular disease SNP data. Three out of 15 genes had been reported to be associated with cardiovascular disease: *HLA-DRB1*, *LRP1*, and *MMP2*. We defined these genes as key diabetes-associated susceptibility genes for cardiovascular disease. The expression level of the key genes is shown in Figure 9. *LRP1* and *MMP2* were upregulated in T2DM arterial tissue, while *HLA-DRB1* was downregulated in T2DM arterial tissue.

## 4. Discussion

Cardiovascular disease is a serious complication of DM. Clinically, patients with cardiovascular disease and DM generally have a poorer prognosis compared with the patients without DM. Moreover, decreasing the rates of cardiovascular events has proved to be more difficult than simply intensifying the management of hyperglycemia. The molecular mechanisms involved in the pathophysiology of cardiovascular disease in patients with DM remain unclear. Therefore, exploring susceptibility modules and genes for cardiovascular disease in diabetic patients is essential.

In this study, we built the coexpression modules via WGCNA using the published data. We identified key modules in the blood vessels of diabetic patients compared with those of nondiabetic patients' blood vessel. The function enrichment was investigated. Finally, *HLA-DRB1*, *LRP1*, and *MMP2* were identified as key genes.

WGCNA is a systems biology method for describing the pairwise relationships among gene transcripts [8]. Compared with those bioinformatics articles that only analyzed the differentially expressed genes, our work required a high-power computer and carefully distinguished the false-positive results. Its merit is obvious: the results would be more complete. To our knowledge, this was the first study that used the WGCNA method to construct a coexpression network to explore diabetes-associated susceptibility modules and genes for cardiovascular disease.

By deeply and systemically reanalyzing the GSE13760 dataset, we identified the greenyellow module as being significantly relevant to cardiovascular disease in patients with diabetes. GO analyses demonstrated that calcium ion binding was activated during the development of cardiovascular disease in diabetic patients. Studies have shown that calcium ion binding is crucial in the process of arterial calcification and atherosclerosis [11]. KEGG analyses demonstrated that antigen processing and presentation, phagosome, were important pathways in this context. Antigen processing and presentation, phagosome, are closely associated with autophagy, which is associated with the pathogenesis of diabetes [12]. Dysregulation of autophagy frequently leads to atherosclerosis [13]. The physiological process of antigen processing and presentation, phagosome, may provide potential targets for the prevention of or intervention in cardiovascular disease in diabetic patients.

Here, we identified three diabetes-associated susceptibility genes for cardiovascular disease: *HLA-DRB1*, *LRP1*, and *MMP2*. The human leukocyte antigen (HLA) complex is a gene family that is involved in antigen presentation associated with protection against, or susceptibility to inflammatory, infectious and autoimmune diseases. The study reported by Williams found lower expression of the HLA-DRB1 mRNA in type 2 diabetes, which was consistent with our findings, and suggests that HLA-DRB1 is protective for type 2 diabetes by increasing insulin secretion [14]. There is some evidence of the potential role of HLA in the pathogenesis of diabetic complications. The study reported by Marzban found a potential protective role for the HLA-DRB1^∗^07-DQB1^∗^02 haplotype against the development of peripheral neuropathy in patients with T2D [15]. The study of Cordovado et al. showed that carriers of DRB1^∗^04 were protected against the injurious hyperglycemic effects related to nephropathy in type 1 diabetes [16]. Our findings demonstrated a potentially protective role of HLA-DRB1 against the development of cardiovascular disease in patients with T2D. Atherosclerosis, which is the key pathophysiology of cardiovascular disease, is a chronic inflammatory disease in which HLA molecules play a role in the initiation and development of the condition. Golmoghaddam et al. reported a significantly lower frequency of HLA-DRB1^∗^01 in patients with coronary artery atherosclerosis, suggesting that HLA-DRB1^∗^01 is a protective allele against atherosclerosis, which is consist with the results of our study [17]. A genome-wide meta-analysis showed *HLA-DRB1* was associated with human longevity [18]. Other studies showed various *HLA-DRB1* alleles could contribute differently to susceptibility of cardiovascular disease [19, 20].

The LDL receptor-related protein 1, which is encoded by *LRP1*, is a large endocytic and signaling receptor [21]. An animal study showed that LRP1 expression was increased in cardiomyocytes isolated from acutely diabetic rats [22]. Other studies reported the upregulation of LRP1 in epicardial fat and skin from diabetic patients compared with control individuals, which was consistent with our findings [23, 24]. In contrast, another study showed a significant reduction in LRP1 expression in subjects with diabetic peripheral neuropathy compared with the noncomplication group [25]. Highly diverse functions of LRP1 have been reported in different tissues. The current view of the role of LRP1 in atherosclerosis formation is controversial. LRP1 plays a crucial role in the dysregulated cholesterol transfer from modified lipoproteins to human coronary vascular smooth muscle cells. The study reported by de Gonzalo-Calvo [26] showed that the circulating soluble LRP1 levels were increased in the conditioned medium of coronary atherosclerotic plaque areas extracted from patients compared with nonatherosclerotic areas of the same coronary artery and patient. In contrast, Mueller's study showed that the absence of LPR1 accelerates atherosclerosis regression in macrophages, indicating an atheroprotective role for LPR1 [27]. Recent studies revealed that LRP1 regulates the insulin signaling pathway directly [28–31]. LRP1 potentially plays a crossroad role between diabetes and cardiovascular disease. To date, no study has directly demonstrated the role of LRP1 in diabetic vascular disease. Further investigation is necessary to clarify the underlying biological pathways.

MMP-2 is a protease that degrades extracellular matrix components in normal and pathological conditions. MMP2 was significantly increased in patients with diabetes and in women with metabolic syndrome, which was consistent with our findings [32, 33]. Other studies suggested a potential role for MMP2 in the pathogenesis of diabetic complications. The study reported by Chung et al. showed that MMP-2 was upregulated in the arterial vasculature of CKD patients with diabetes and was correlated with arterial stiffening, impaired angiogenesis, and endothelial dysfunction [34]. Several studies have shown that MMP-2 is a key molecule in diabetic retinopathy [35–38]. Moreover, increased plasma MMP-2 was associated with macrovascular disease by increasing vascular remodeling [39, 40]. Peters et al. [41] reported that higher levels of MMP-2 are associated with cardiovascular disease in type 1 diabetes. As an important contributing factor to the development of vascular lesions, MMP-2 may play an important role in the high susceptibility to CVD observed in diabetic patients. Deep investigation of *MMP2* in cardiovascular disease in patients with type 2 DM is required.

This study had several limitations. A larger sample size will be needed in future studies. Moreover, functional studies of the module and key genes identified here are needed. Finally, methods based on molecular biology approaches should help validate our findings.

## 5. Conclusion

This was the first study that used the WGCNA method to construct a coexpression network to explore diabetes-associated susceptibility modules and genes for cardiovascular disease. Our findings revealed a module and several key genes that acted as essential components in the etiology of diabetes-associated cardiovascular disease, which may enhance our fundamental knowledge of the molecular mechanisms underlying this disease.

## Figures and Tables

**Table 1:** Hub genes of the module greenyellow. Hub genes were defined as having a gene significance over 0.2 and a module membership over 0.8.

Gene	Gene significance	Module membership
*SEPT11*	0.39	0.88
*AKR1C1*	0.43	0.89
*CD74*	0.25	0.89
*CST3*	0.37	0.88
*DCN*	0.37	0.86
*FBLN1*	0.51	0.88
*HLA-DRB1*	0.22	0.83
*LRP1*	0.50	0.82
*MMP2*	0.39	0.92
*MRC2*	0.23	0.82
*NPC2*	0.21	0.85
*PCDHGA1*	0.44	0.80
*PRELP*	0.55	0.84
*SPTBN1*	0.42	0.81
*TGFBR2*	0.23	0.88

## Data Availability

The raw data used to support the findings of this study are freely available from GEO datasets GSE13760.
